# Therapeutic Irradiation: Consequences for Bone and Bone Marrow Adipose Tissue

**DOI:** 10.3389/fendo.2019.00587

**Published:** 2019-08-29

**Authors:** Samantha Costa, Michaela R. Reagan

**Affiliations:** ^1^Center for Clinical and Translational Research, Maine Medical Center Research Institute, Scarborough, ME, United States; ^2^University of Maine Graduate School of Biomedical Science and Engineering, Orono, ME, United States; ^3^Tufts University School of Medicine, Boston, MA, United States

**Keywords:** irradiation, bone marrow microenvironment, bone marrow adipose tissue, osteoblast, adipocyte

## Abstract

Radiotherapy continues to be one of the most accepted medical treatments for cancer. Localized irradiation is the most common treatment for prostate, pancreatic, rectal, cervical and endometrial malignancies. Conventional localized fractions are total doses of 30-62Gy at 1.8-2Gy per fraction, with administration of ~60Gy often used for tumor ablation. However, even the lowest dose of localized irradiation exposure can result in adverse complications to adjacent organs, tissues, and vessels, which absorb a portion of the treatment. Skeletal complications are common amongst cancer patients undergoing these localized treatments. Irradiation exposure causes deterioration to the overall quantity and quality of bone by interfering with the trabecular architecture through increased osteoclast activity and decreased osteoblast activity. Irradiation-induced bone damage parallels adipocyte infiltration of the bone marrow (BM) resulting in compositional alterations of the microenvironment that may further affect bone quality and disease state. There may also be direct effects of irradiation on the BM adipocyte/pre-adipocyte, although *in vitro* findings do not always agree and cellular response is dependent on irradiation dosage. Hematopoietic cells also become apoptotic upon irradiation, which causes a range of skeletal effects. Bone loss leaves patients at a greater risk for osteopenia, osteoporosis, osteonecrosis, and skeletal fractures that drastically reduce quality of life. Osteoanabolic agents stimulate bone formation and reduce fracture risk in patients with low bone density; thus, osteoanabolic or anti-resorptive agents may be useful co-treatments with irradiation. This review discusses these topics and proposes further research directions using novel or combination therapies to enhance bone health during irradiation.

## Introduction

Since the discovery of the X-ray in 1895, irradiation science has offered advanced developments in techniques, multidisciplinary approaches, and research (1). Radiation therapy continues to be a widely accepted treatment for malignant cancers through its effective manner of killing cancer cells and reducing tumor size (1, 2). Irradiation induces free radicals in the form of reactive oxygen species (ROS) that leads to DNA damage (2). With improved treatment regiments, there have been improvements in disease outcomes and reductions in the adverse side effects of irradiation therapy (1, 2). The effectiveness of irradiation therapies, in combination with advances in pharmaceutical treatments, early detection, prevention, and cancer awareness, has drastically improved patient quality of life and decreased mortality rates; in some cases, these advancements have changed cancer from being an acute disease to a treatable chronic disease (1). The American Cancer Society projected there were over 2.6 million fewer cancer-related deaths from 1991 to 2016 (3).

Despite the advancements in irradiation therapies, there is still an unmet concern surrounding the systemic and localized effects of irradiation on adjacent tissues, vessels, and bone (4–6). Patients undergoing irradiation therapy have the potential to experience increased irradiation toxicity, even with fractionation of treatments, and adjacent soft tissue and bone damage as an adverse side effect due to limited tumor uptake and retention of irradiation doses (6). Tumor microenvironments promote tumor growth and angiogenesis through paracrine stimulatory factors and immune-mediated interactions (7). There are many cytokines released by the immune system that are considered “pro-tumor” or “anti-tumor” that alter the tumor microenvironment (7). After irradiation exposure, there are increased inflammatory cytokines, IL-1α/β, IL-6, IL-17, TNF-α, and VEGF, and evidence of increased cellular senescence, demonstrated through increased senescence-associated secretory phenotype (SASP) proteins (2, 7, 8). This change to the tumor microenvironment and increased immune activity is thought to explain the abscopal effect, in which localized irradiation results in regression of metastatic cancer that are distant from the initial irradiation site (7).

Due to the high calcium content, bone absorb 30-40% more irradiation than the surrounding tissues; thus, the absorption of any given irradiation dose is considerably higher in bone than the surrounding tissues, making bone a common site for irradiation-induced damage (9). As previously discussed, irradiation exposure releases cytokines as an injury response that triggers acute inflammation (9, 10). This acute inflammation is characterized by increased vascular permeability with localized edema, destruction of endothelial cells, and an association with vascular thrombosis (9). Irradiation exposure also induces late stage fibroatrophy that results in poorly vascularized tissue which does not allow for proper healing, ultimately increasing tissue fragility and the recurrence of inflammation upon local injury (9, 10).

**Figure 1:**
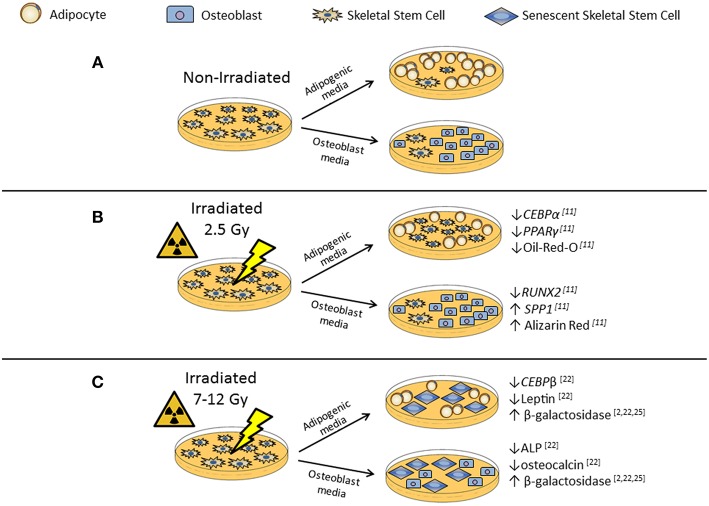
*In vitro* irradiation affects adipogenic and osteogenic differentiation potential of skeletal stem cells (SSCs). **(A)** Non-irradiated SSCs, represent control differentiation. **(B)** Low-dose (2.5Gy) irradiated SSCs in adipogenic or osteogenic differentiation media have differing differentiation potentials. Low-dose irradiation caused reduced adipocyte differentiation with decreased adipocyte markers, such as *CEBP*α and *PPAR*γ, and decreased Oil-Red-O staining when compared to the control. Osteoblast differentiation showed decreased *RUNX2* expression, but increased mineralization markers *SPP1* and Alizarin Red staining after low-dose exposure when compared to the control. There was no significant difference in osteoblast differentiation when compared to the controls. **(C)** High-dose (7-12Gy) irradiated SSCs have reduced adipocyte and osteoblast differentiation potential and evidence of increased β-galactosidase activity, a marker for cellular senescence.

The damage observed within the bone and bone marrow (BM) after irradiation therapy is similar to the pathological conditions seen with osteoporosis (2). There is a decrease in trabecular bone volume, an increase in bone marrow adiposity (BMA), increased CTX/TRAP5 levels in the serum, and prolonged fracture healing times (2). Irradiation also depletes hematopoietic and skeletal stem cell populations within the BM (11–13). Bone marrow transplants allow for trabecular recovery, reduced BMA, and increased cell number within the BM microenvironment (12). Skeletal stem cells (SSCs), previously referred to as mesenchymal stem cells, appear to be effected by the irradiation source (photon irradiation vs. ionizing irradiation) and dose resulting in the varying differentiation potential observed in different *in vitro* studies (Figure 1) (11, 14–16). In murine models, the balance favors adipogenesis at the expense of osteogenesis, as a result of irradiation-induced bone loss. This bone loss is in part due to the increased osteoclast activity immediately following irradiation exposure and then the latent decrease in osteoblast activity in the sequential weeks (4, 5, 12, 17).

Studies have shown that osteoanabolic agents stimulate bone formation and reduce fracture risk in patients with low bone density (18–20). Since an adverse side effect of irradiation is decreased bone density and increased bone fragility, combination therapies of osteoanabolic, or anti-resorptive agents may be useful for patients receiving irradiation therapies. This review will discuss these topics and propose further research directions including *in vitro* and *in vivo* studies using novel or combination therapies to enhance bone strength in patients after irradiation (18).

## *In vitro* Irradiation of Skeletal Stem Cells Alters Differentiation Potential

The regenerative capabilities of SSCs *in vitro* have been shown in a multitude of tissue damage models (14). Human bone marrow skeletal stem cells (hSSCs) have been shown to be resistant to the effects of low-dose irradiation (2.5Gy) with no apparent changes to morphology or immunophenotype (11). Preciado et al. have shown irradiated and non-irradiated hSSCs still expressed CD73, CD90, CD105, CD44, and CD166 and are negative for CD34, CD45, CD14, CD19, and HLA-DR, which are definitive markers of a typical SSC immunophenotypic profile (11). Irradiated hSSCs also had no significant changes to cell viability 1 or 72 h after exposure compared to non-irradiated hSSC controls (11). However, low-dose irradiation exposure affected hSSC behavior and differentiation potential *in vitro* (11). Irradiated hSSCs were capable of differentiation, but had significantly less adipocytes, evident through decreased Oil-Red-O staining and significantly less mRNA expression of adipogenic differentiation markers, *CEBP*α and *PPAR*γ, when compared to the non-irradiated cells (11). On the other hand, low level irradiation exposure stimulated hSSCs osteogeneic differentiation apparent through increased mineralization expression of *SPP1* and Alizarin Red staining, despite having reduced *RUNX2* expression, an early osteogenesis marker (11).

The observed *in vitro* results in the Preciado study are likely because the exposure was a single, low-dose compared to other studies that use a single high-dose or fractionized doses that are needed therapeutically (4, 5, 17, 21, 22). Other studies suggest SSC retention of stem cell characteristics is dose-dependent and can be altered with a single high-dose exceeding 10Gy (22–24). Schönmeyr et al. demonstrated the effects of high-dose irradiation (7 and 12Gy) on rat SSCs (rSSCs) resulted in a dose dependent response compared to non-irradiated cells (22). After high-dose irradiation exposure rSSCs had a higher percentage of apoptotic cells and more cells in the G_2_ cellular arrest phase (22). The irradiated cells also had reduced expression of osteogeneic markers, *ALP* and osteocalcin, as well as reduced expression of adipogenic markers, *LPL, CEBP*β, and Leptin (22). The reduced differentiation potential seen *in vitro* could be evidence of irradiation-induced cellular senescence (2, 11). A marker for senescence, β-galactosidase, has been used *in vitro* to show irradiation-induced senescence in a time and dose dependent manner (2, 22, 25). The level of differentiation potential down the osteogenic and adipogenic lineages of SSCs has been shown to be more sensitive or more resistant based on the dosage of ionizing radiation (11, 22, 26, 27). The altered SSC differentiation capacity impacts the hematopoietic niche and enhances engraftment of BM derived stem cell transplantations to the BM microenvironment (12).

Interestingly, irradiation induced by radionucleotides, such as Strontium-90, can also induce similar effects on SSCs (15). After 7 days of exposure *in vitro*, a pre-osteoblast cell line showed a decreased ability to proliferate, changes in cytokine expression, and changes in their ability to support hematopoietic progenitor proliferation and differentiation (15). Exposure to Strontium-90 also showed evidence of increased senescence through increased β-galactosidase as well as senescent morphology with enlarged cytoplasm and nucleus (15). Despite these intriguing findings *in vitro, in vivo* studies are necessary to determine if the same *in vitro* phenomena are observed.

### *In vivo* Irradiation in Rodents (Mouse and Rat) Cause Bone Loss and Increased BMAT

**Figure 2:**
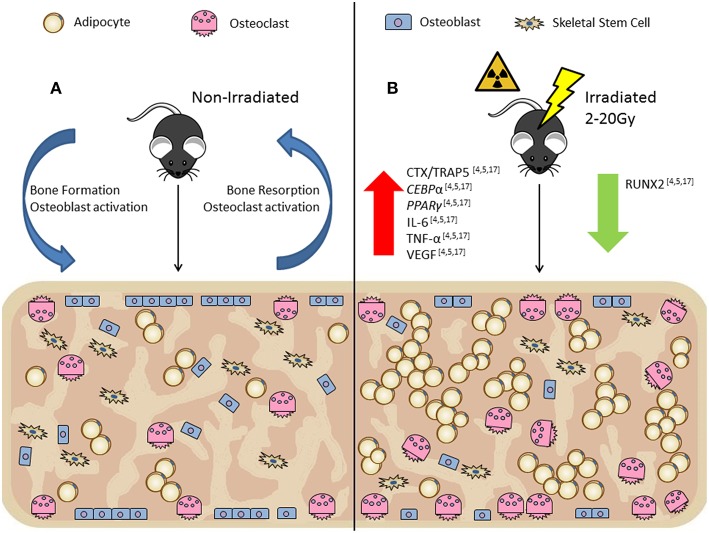
*In vivo* rodent models show irradiation alters the bone marrow microenvironment by increasing osteoclast numbers per bone surface (Oc.S/BS) and decreasing osteoblast numbers per bone surface (Ob.S/BS) resulting in decreased trabecular bone volume with a rapid influx of bone marrow adipocytes. **(A)** Non-irradiated control, demonstrates normal bone turnover processes. **(B)** Irradiated (2-20Gy), demonstrates the uncoupling of the bone formation/resorption ratio through increased CTX/TRAP5 (osteoclast activity) and decreased *RUNX2* (osteoblast activity) expression. *In vivo* irradiation exposure also has increased *CEBP*α and *PPAR*γ (adipogenesis markers), and IL-6, TNF-α, and VEGF (inflammatory and senescent markers).

In contrast to the decreased adipogenesis induced by irradiation observed in *in vitro* studies, *in vivo* models demonstrated that irradiation increases BMA and deteriorates trabecular bone at both high and low irradiation doses. For example, Willey et al. showed that as early as 3 days post low-dose (2Gy) whole-body irradiation of thirteen-week-old C57BL/6 mice there was a significant increase in osteoclast activity through significantly increased TRAP-5b serum levels and significantly increased osteoclast numbers per bone surface, although BMA analysis was not done (21). Ten days post low-dose (5Gy) whole-body irradiation exposure of C57BL/6 mice resulted in rapid infiltration of BM adipocytes within the BM (5). This significant increase in BMA was coupled with a significant decrease in trabecular bone volume/total volume (BV/TV), which has been observed in 8 and 16-week-old C57BL/6 mice (5, 17). This irradiation-induced bone damage was not recovered 8 weeks post exposure (5, 17). BM recovery of 8 and 16-week-old mice has been shown to be age and time dependent (17). Two and ten-days post irradiation exposure, the total number of BM cells in 8 and 16-week-old mice were significantly decreased by more than 60% (17). By 8 weeks, only 8-week-old mice showed recovery to their BM cells (17). In sum, *in vivo* models using a wide range of irradiation doses have consistently shown that irradiation decreases trabecular bone volume and increases BMA compared to non-irradiated controls (Figure 2). This finding is likely due to a shift in SSC lineage differentiation (i.e., favoring adipogenesis over osteogenesis) that appears to be in response to irradiation-induced BM microenvironment alternations, rather than SSC autonomous responses to irradiation, because these same shifts are not observed in SSCs *in vitro*.

Localized irradiation has been shown to affect healthy tissue adjacent to the irradiation site, with about half of the localized dose being absorbed by healthy tissue and bone (4). In 4 month old male Sprague-Dawley rats, localized exposure of 20Gy to the right hind-limb, spanning the proximal tibia to the distal femur, revealed significant reductions in trabecular bone mineral density (tBMD) and trabecular BV/TV of the irradiated femur and also in the contralateral femur compared to the sham irradiated controls (4). Cortical thickness was not affected by irradiation, but cortical porosity was increased in the irradiated and contralateral femur (4). BMAT increased at 2 weeks and 12 weeks post-irradiation in the irradiated and contralateral tibias. These alterations in trabecular bone were due to a decrease in osteoblast surface per bone surface at 2 and 12 weeks post-irradiation in the irradiated and contralateral tibias (4). Within the BM, expression of *RUNX2* and *PPAR*γ of osteoblast and adipocyte progenitor cells were determined using reverse transcriptase-PCR (RT-qPCR) (4). At 2 weeks, *RUNX2* and *PPAR*γ expression were significantly decreased in both the irradiated and contralateral (4). By 12 weeks, the mRNA expression of *RUNX2* continued to be downregulated by 94.5% in the irradiated and 44.1% in the contralateral, yet the expression of *PPAR*γ was upregulated by 13-fold in the irradiated and 9-fold in the contralateral relative to the control (4). This study demonstrated how irradiation-induced bone damage does not require direct exposure to result in impairment, referred to as the bystander effect (4, 28).

There is also a rapid increase in osteoclast activity after irradiation exposure, seen with an increase in osteocalcin and TRAP5 levels in rat serum (4). However, 2 weeks post-irradiation there were no significant effects on bone suggesting irradiation did not compromise or uncouple the bone formation/resorption ratio immediately after exposure (4). These results differ from the observed changes seen in many irradiation mouse models. By 12 weeks post-exposure, there were significant decreases in trabecular bone volume, yet osteoclastogenesis was now comparable to the controls while osteoblastogenesis was significantly decreased, resulting in an altered formation/resorption ratio within the BM microenvironment that affected bone quality (4).

### Clinical Trials Mirror *in vivo* Animal Model Findings

Irradiation-induced bone loss has been reported to cause more than insufficiency fractures; other complications from irradiation therapy include osteitis and osteolysis (29). A patient study of 510 patients (ages 40–84 years) analyzed pelvic bone related complications after irradiation therapy for uterine cervical cancer. Osteolysis was detected in 4 patients and avascular necrosis of the femoral head was diagnosed in 2 patients post irradiation therapy (29). One-hundred patients were diagnosed with insufficiency fractures a median of ~16.9 months (range 1–87 months) after pelvic irradiation therapy (29). Of the patients diagnosed with insufficiency fractures, 85% had sacral involvement and 61% developed multiple pelvic insufficiency fractures; 40% of those patients had symmetric bilateral lesions of the sacral alae (29).

Patients can experience late stage complications from irradiation exposure as part of an advanced treatment for head and neck tumors (9). Osteoradionecrosis (ORN) of the jaw bones (and surrounding soft tissue) is the most severe last stage complication (9). ORN illustrates increased inflammation and the development of hypovascular, hypocellular, and hypoxic tissues, which causes increased cell death and collagen breakdown that exceeds the normal cell repair and collagen synthesis homeostasis (9, 30). ORN diagnostic criteria is a slow healing (failure to recover over a 3 month period) irradiation-induced necrosis of the bone, associated with surrounding tissue necrosis in the absence of local tumor necrosis, recurrence, or metastatic disease (9). Irradiated specimens were obtained from 40 patients treated for ORN (control specimens were obtained from non-irradiated patients treated from head and neck tumors) (9). The total irradiation dosage of these specimen ranged from 50.4 to 70.4Gy (9). A histopathology examination on the bone and soft tissue samples revealed hyperemia and endarteritis as early effects of irradiation that were prolonged for up to 6 months post exposure (9). Signs of increased hypocellularity occurred rapidly after irradiation exposure; the irradiated bone samples showed greater cell loss than the soft tissue samples (9, 31). Evidence of thrombosis was apparent through densely fibrous material seen years post irradiation exposure (9, 31). There was a loss in vascular content, increase in BMAT, and fibrosis that showed a linear correlation to the time post exposure that were considered end stage markers of the irradiation-induced injury (9, 31). This current study suggested the increase in BMAT in the irradiated bone samples was due to the stunted bone turnover processes (9, 32).

Another clinical study showed 13 female patients, ages 35–63 years old, with gynecological malignancies that received irradiation or chemotherapy treatments had increased BMAT 6 and 12 months after initiating therapy treatments through repeated MRI scan (baseline, 6 months, and 12 months post therapy initiation) (33). Sagittal images of signal fat fraction (SFF) were taken in patients receiving focal irradiation therapy with the pelvic region as the target field (33). Approximately half of the irradiation therapy dose was absorbed by the sacrum and adjacent tissues and bones, such as the L4 vertebral body (33). At the baseline MRI scan, the SFF of the BMAT in the L4 and S1 were similar, however, by the 6-month scan the increase in the SFF in the S1 was marginally higher than in L4 (33). The increased SFF seen in S1 compared to L4 correlated to the higher irradiation absorption at S1 resulting in more BMAT accumulation (33). It is believed there is a progressive conversion of the hematopoietic marrow to the more adipocyte rich, yellow marrow observed throughout these regions as a response to irradiation treatment (33). The SFF of the L4 and femoral neck increased at the 6-month post-treatment scan (irradiation and chemotherapy treatments combined in this analysis) (33). These significant increases of SFF in the L4 and femoral neck were compared to skeletal muscle and subcutaneous white adipose tissue as controls, demonstrating the effects of irradiation on localized tissues and more specifically on the BM microenvironment (33).

### Bisphosphonates and Osteoanabolic Agents Have Potential Restorative Effects on Irradiation-Induced Bone Damage

Anti-resorptive agents, such as bisphosphonates (BPs), and osteoanabolic agents are commonly used as osteoporosis treatments. BPs mediate bone resorption through osteoclast apoptosis (19, 20, 34). BPs can also reduce osteoblast and osteocyte apoptosis, but do not actively result in bone accrual (34). In rodent models, the administration of BPs following irradiation therapy can improve bone quality, bone strength, and BMD (19, 35, 36). However, there are conflicting data across patient trials regarding bone quality and pain management with BPs alone, in respect to cancer and irradiation therapies, suggesting combination treatments of BPs, and osteoanabolic agents may be needed to combat irradiation-induced bone damage (20, 35, 37–39).

Well-studied osteoanabolic treatments are human parathyroid hormone (hPTH) and sclerostin antibody (Scl-Ab). Both of these agents stimulate bone formation, but through different modes of action (40, 41). Scl-Ab increases bone formation by inhibiting sclerostin binding to lipoprotein receptor protein (LRP) 5/6 that inhibits canonical WNT signaling and subsequently activating SSC differentiation into osteoblasts (40, 41). Scl-Ab also suppresses bone resorption through inhibitory effects on osteoclastogenesis regulators (41–43). hPTH stimulates both anabolic and catabolic activities with a net gain in favor of the former, but there is evidence that hPTH anabolic capabilities are dependent on its ability to stimulate osteoclastogenesis (41, 44). Not only do these osteoanabolic agents increase bone accrual, but they also decrease BMA (41). Scl-Ab results in increased trabecular bone and decreased BMA, but hPTH has a direct effect on BMA reductions despite bone accrual (41). Since irradiation causes bone deterioration and adipocyte infiltration, which in combination may exacerbate bone related complications, administration of osteoanabolic agents in conjunction with irradiation therapy may prevent excessive bone loss and adipocyte infiltration (16).

## Conclusion

Clinical studies and *in vivo* rodent models have shown high-dose and sub-lethal dose irradiation causes rapid bone loss due to increased osteoclast activity and decreased osteoblast activity, which results in increased BMA and secondary late stage bone complications that are believed to be from continued irradiation damage (16). Through *in vitro* experiments, it has been shown that SSCs are capable of maintaining their proliferation, differentiation, and regenerative properties at low-dose irradiation exposure, but at a lower capacity than non-irradiated SSCs (11). However, after high-dose exposure SSCs lose their stem cell characteristics or experience cell death (22–24, 26, 27). Currently, irradiation therapy is provided to patients in fractionized doses, but even low-doses over the course of several weeks show signs of decreased BMD and increased BMA (16). With decreased BMD, patients are at a greater risk for skeletal fractures and other bone related diseases and complications. A patient's quality of life is severely affected by these irradiation-induced bone incidences. Future directions point to more investigative research (and potential clinical practices) into the benefits of combination therapies to reverse the adverse side effects of irradiation-induced bone loss and adipocyte infiltration. Osteoanabolic agents, such as Scl-Ab or hPTH, and BPs may be needed in conjunction or following irradiation therapy treatments to combat the bone, tissue, and cell damages currently being observed.

## Author Contributions

SC wrote the manuscript. MR advised and edited the manuscript. MR and SC approved the final version of the manuscript.

### Conflict of Interest Statement

The authors declare that the research was conducted in the absence of any commercial or financial relationships that could be construed as a potential conflict of interest.
